# News and Coming Events

**Published:** 1908-05-16

**Authors:** 


### NEWS AND COMING EVENTS.

Dr. D. MacAlister, Principal of the University of Glasgow, has announced his intention to resign his office as representative of the University of Cambridge on the General Medical Council at the close of this month.

The annual meeting of the Rural Midwives Association will be held (by permission of the Lady Esther Smith), at 3 Grosvenor Place, S.W., on May 27, at 3 o'clock, Mr. F. Newman Rogers, M.P., in the chair.

Professor David W. Finlay, M.D.Glasg., LL.D., F.R.C.P.Lond., will preside at a dinner of the Glasgow University Club, London, at the Trocadero, Piccadilly Circus, London, W., on Friday, May 29.

The President of the Queen Victoria Memorial Hospital, Nice (Sir George White), has received an intimation from Mr. John Jaffe, of Belfast and Nice, that he intends to defray the whole cost (25,000 fr.) of the new wing of the hospital.

The Croonian Lectures before the Royal College of Physicians of London will be delivered on Tuesdays and Thursdays, June 18 to 30, by Dr. A. E. Garrod, at 5 p.m. on each day. The subject of the course is "Inborn Errors in Metabolism."

King Edward has appointed Dr. Egeberg, Physician in Ordinary to the King of Norway, to be a Commander of the Royal Victorian Order; and King Haakon has conferred the Grand Cross of the Order of St. Olav upon Sir Francis Laking.

The fifth Welsh medical dinner will be held at the Criterion Restaurant, London, on Friday, May 29, at 7.30 p.m. Mr. Edmund Owen will be in the chair. The Honorary Secretary is Mr. J. Howell Evans, M.Ch., F.R.C.S., 25 Berkeley Square, London, W.

Mr. A. W. West, Treasurer and Chairman of St. George's Hospital, has written to the Press contradicting recent unauthorised and inaccurate statements as to the removal of St. George's Hospital from its present site. He states that no question relative to the removal or otherwise of the Hospital has been recently before the Governors.

The King of Italy has conferred upon Dr. Thomas Reid, Honorary Consulting Oculist to the Western Infirmary and Sick Children's Hospital, Glasgow, the distinction of "Commendatore of the Crown of Italy" in recognition of his merit as an ophthalmic surgeon and of his special services to the Clinic of Ophthalmology in the University of Turin.

A conversazione will be held at the rooms of the Medical Society of London, 11 Chandos Street, Cavendish Square, London, W., on Monday, May 18, at 8.30 p.m. The Fothergillian medal will be presented by the President of the Society to Sir Almroth Wright, F.R.S., at 8.45 p.m. At 9 o'clock Mr. Clinton T. Dent will deliver an oration on "The After-Results of Injuries."

A conference on capital punishment, to be held in Caxton Hall on Thursday afternoon, June 18 next, is being arranged by the Romilly Society, with the co-operation of the Medico-Legal Society, the Society for the Abolition of Capital Punishment, the Penal Reform League, etc., and prominent speakers have promised to take part.

According to a Reuter's telegram of May 12, Dr. Raikes and Dr. Wray, Government medical officers at Singapore, have died of plague contracted while performing a post-mortem examination on a patient who died while in quarantine.

Mr. Alexander John Balmanno Squire, M.B.Lond., the well-known dermatologist, died on May 7 at 24 Weymouth Street, at the age of 71. He was educated at University College Hospital and qualified in 1858, and subsequently took the M.B. degree at London University (gold medallist). He had been senior surgeon to the British Hospital for Diseases of the Skin, medical officer of St. Marylebone General Dispensary, and lecturer at St. Mary's Hospital Medical School. He was the author of several works on diseases of the skin.

Miss Ellen Culpan, of Nunroyd, Heckmondwike, Yorkshire, who died on March 16 last, bequeathed £1,350 each to the Leeds General Infirmary, the Bradford Royal Infirmary, the Royal Halifax Infirmary, the Royal Albert Asylum at Lancaster, the Bradford Children's Hospital, the Bradford Eye and Ear Hospital, the St. Catherine's Home at Bradford, and the Yorkshire Home at Harrogate for Chronic and Incurable Diseases; and £500 each to the Batley and District Hospital, and the Dewsbury and District General Infirmary.

An examination of candidates for thirty commissions in the Royal Army Medical Corps will be held on July 29 and following days. Applications should be made to the Secretary, War Office, London, not later than July 20. Candidates who are over the regulated limit of age at the date of the examination will be permitted to deduct from their actual age any period of service in the field after October 1, 1899, that they could reckon towards retired pay and gratuity, if such deduction will bring them within the age limit. The presence of candidates will be required in London from July 27.

In response to the desires of a number of School Medical Officers, arrangements are being made for a short course of lectures and demonstrations to be given in London during Whit week. Dr. James Kerr, Dr. H. Meredith Richards, Dr. C. J. Thomas, and other experienced medical inspectors have already promised to give addresses, and demonstrations of the details of actual medical inspection will also be arranged. School Medical Officers desiring to avail themselves of the course are requested to communicate with Mr. Wm. A. Lawton, at the offices of the Incorporated Society of Medical Officers of Health, 1 Upper Montague Street, Russell Square, London, W.C.

The Governors of the Royal Hants County Hospital, owing to lack of funds, have decided to reduce the number of beds in actual regular use to ninety-two, with eight more reserved for emergency cases; and the out-patient department is to be closed to all except special cases requiring treatment by *x*-rays, Finsen light, and the like. The managing committee have now been authorised to take payments from patients, the straitened circumstances of the institution having induced the Governors reluctantly to depart from a rule which has prevailed ever since the foundation of the hospital, nearly 170 years ago. Of late years the income has remained the same, but the number of patients has almost doubled.

The *London Gazette* announces that Inspector-General of Hospitals and Fleets Sir H. M. Ellis, K.C.B., K.H.P., F.R.C.S., LL.D., Director-General of the Medical Department of the Navy, has been placed on the Retired List at his own request.

A performance of "Veronique," in aid of the Lord Mayor's special appeal for the funds of the London Hospital, will be held at the Scala Theatre, Charlotte Street, Fitzroy Square, W., on Thursday, Friday, and Saturday, May 21, 22, and 23, at 8 p.m.

The King has been pleased to approve of the retention of the title of "Honourable" by Sir William Bisset Berry, M.D., on his retirement from the office of Speaker of the House of Assembly of the Colony of the Cape of Good Hope, which he has held for a period of more than three years.

The King has given permission to F. Dixon, Esq., to accept and wear the insignia of the Second Class of the Imperial Ottoman Order of the Medjidieh (being a promotion in that Order), and to A. Granville, Esq., M.R.C.S., L.R.C.P., to accept and wear the Insignia of the Fourth Class of the same Order, these decorations having been conferred upon them by his Highness the Khedive of Egypt, authorised by his Imperial Majesty the Sultan of Turkey, in recognition of valuable services rendered by them.

The next general meeting of the Medico-Psychological Association of Great Britain and Ireland will be held at 11 Chandos Street, Cavendish Square, London, W., on Tuesday, May 19, under the presidency of Dr. P. W. Macdonald, at 3 p.m. A paper on the "Histology of Lymphogenous and Hæmatogenous Toxic Lesions of the Spinal Cord" will be read by Dr. David Orr and Dr. R. G. Rows. A discussion will be introduced by Dr. D. G. Thomson on the "Teaching of Psychiatry." Members will afterwards dine together at the Café Monico at 7 o'clock.

The Vice-Chancellor of the University of Oxford has received an intimation from Sir William Selby Church, D.M., D.Sc., Hon. Fellow of University College, that the trustees of the fund collected by the medical graduates in London have paid over the residue of the fund, amounting to somewhat over fifteen hundred pounds, to the trustees of Lord Curzon's Endowment Fund, on condition that the said sum shall be preserved as capital for the benefit of the University and the interest shall be employed for some purpose connected with the Department of Pathology in the University.

Dr. Charles James Cullingworth, M.D., F.R.C.P., consulting obstetric physician St. Thomas's Hospital, died on May 11, in his sixty-seventh year. He was educated at Wesley College, Sheffield, and at the Leeds School of Medicine, and qualified in 1865. After being lecturer and professor at Owens College, Manchester, and on the staff of St. Mary's Hospital, Manchester, he became, by invitation, senior obstetric physician at St. Thomas's Hospital, London (1888-1904), and held other important medical appointments in London. Dr. Cullingworth received the honorary degree of LL.D. from Aberdeen and of D.C.L. from Durham University. He was elected President of the Obstetrical Society of London in 1897, and a member of the Central Midwives Board in 1903. He was a fellow of many learned societies in this country and abroad, and until a few years ago he was a frequent contributor to the literature of obstetrics and gynæcology.

The annual meeting of the Governors of the Gordon Hospital for Fistula and other Rectal Diseases was held on May 6, the Earl of Buchan, the President of the hospital, being in the chair. Lord Buchan remarked upon the good work which was done by the hospital, and drew attention to the fact that the institution was being seriously hampered by want of funds.

Since the publication of the President Lord Cromer's letter on behalf of the Research Defence Society, the following names have been added to the list of vice-presidents of the Society: Lord Ludlow (Treasurer of St. Bartholomew's Hospital), Mr. Briton Rivière, R.A., Earl Egerton, the Marquis of Salisbury, Sir William Turner, K.C.B., Lord Fortescue, Sir Thomas Fraser, Sir John Banks, Count Plunkett, and the Bishop of Chester.

